# Supplementary material for: Influence of Socioeconomic Status on the Presence of Obstructive Coronary Artery Disease and Cardiovascular Outcomes in Patients Undergoing Invasive Coronary Angiography
Source: Healthcare (Basel). 2024 Jan 17;12(2):228. doi: 10.3390/healthcare12020228 (PMC10815423; doi:10.3390/healthcare12020228)
Supplement: Supplementary file 1 [file healthcare-12-00228-s001.zip › healthcare-2793264-supplementary.pdf]

Supplementary Table S1. Amount of monthly income criteria for all Korean people and medical aid beneficiary in 2023

Income	Household size						
	**1-person household**	**2-person household**	**3-person household**	**4-person household**	**5-person household**	**6-person household**	**7-person household**
All Korean people	1461	2430	3118	3798	4451	5082	5701
MAB	546	916	1179	1440	1694	1942	2188

Income is denoted in US Dollars. Data source: Republic of Korea Ministry of Health and Welfare website (https://www.mohw.go.kr/menu.es?mid=a10708010300).
